# Portal Vein Pulsatility as a Dynamic Marker of Venous Congestion Following Cardiac Surgery: An Interventional Study Using Positive End-Expiratory Pressure

**DOI:** 10.3390/jcm10245810

**Published:** 2021-12-12

**Authors:** Pierre Huette, Pierre-Grégoire Guinot, Guillaume Haye, Mouhamed Djahoum Moussa, Christophe Beyls, Mathieu Guilbart, Lucie Martineau, Hervé Dupont, Yazine Mahjoub, Osama Abou-Arab

**Affiliations:** 1 Anesthesia and Critical Care Medicine Department, Amiens Hospital University, 80000 Amiens, France; haye.guillaume@chu-amiens.fr (G.H.); beyls.christophe@chu-amiens.fr (C.B.); guilbart.mathieu@chu-amiens.fr (M.G.); martineau.lucie@chu-amiens.fr (L.M.); dupont.herve@chu-amiens.fr (H.D.); mahjoub.yazine@chu-amiens.fr (Y.M.); Abouarab.osama@chu-amiens.fr (O.A.-A.); 2 Anesthesia and Critical Care Medicine Department, Dijon Hospital University, 21000 Dijon, France; pierregregoire.guinot@chu-dijon.fr; 3 Anesthesia and Critical Care Medicine Department, Lille Hospital University, 59000 Lille, France; Mouhamed.MOUSSA@chru-lille.fr

**Keywords:** echography, venous congestion, portal vein pulsatility, cardiac surgery, mechanical ventilation

## Abstract

We aimed to assess variations in the portal vein pulsatility index (PI) during mechanical ventilation following cardiac surgery. Method. After ethical approval, we conducted a prospective monocentric study at Amiens University Hospital. Patients under mechanical ventilation following cardiac surgery were enrolled. Doppler evaluation of the portal vein (PV) was performed by transthoracic echography. The maximum velocity (VMAX) and minimum velocity (VMIN) of the PV were measured in pulsed Doppler mode. The PI was calculated using the following formula (VMAX − VMIN)/(VMax). A positive end-expiratory pressure (PEEP) incremental trial was performed from 0 to 15 cmH_2_O, with increments of 5 cmH_2_O. The PI (%) was assessed at baseline and PEEP 5, 10, and 15 cmH_2_O. Echocardiographic and hemodynamic parameters were recorded. Results. In total, 144 patients were screened from February 2018 to March 2019 and 29 were enrolled. Central venous pressure significantly increased for each PEEP increment. Stroke volumes were significantly lower after PEEP incrementation, with 52 mL (50–55) at PEEP 0 cmH_2_O and 30 mL (25–45) at PEEP 15 cmH_2_O, (*p* < 0.0001). The PI significantly increased with PEEP incrementation, from 9% (5–15) at PEEP 0 cmH_2_O to 15% (5–22) at PEEP 5 cmH_2_O, 34% (23–44) at PEEP 10 cmH_2_O, and 45% (25–49) at PEEP 15 cmH_2_O (*p* < 0.001). Conclusion. In the present study, PI appears to be a dynamic marker of the interaction between mechanical ventilation and right heart pressure after cardiac surgery. The PI could be a useful noninvasive tool to monitor venous congestion associated with mechanical ventilation.

## 1. Introduction

The early detection of venous congestion is strongly recommended during critical-care management to prevent higher mortality and adverse outcomes as well as reduce the length of hospital stay associated with fluid overload [1,2,3].

Central venous pressure (CVP) is the standard measure of venous hypertension but it is an invasive measurement and there is no agreement on the critical value that should be considered in clinical practice [4]. However, high CVP is associated with organ failure [5]. Notably, acute kidney injury (AKI) is associated with high CVP values [6,7]. This observation is based on the cardiorenal syndrome, implying an increase in backward pressure with greater venous resistance to blood circulation. This leads to a reduction in the arteriovenous gradient across organs, which may lead to organ damage [8]. Based on the same rational, mechanical ventilation can lead to an increase in CVP through the interaction between the heart and lungs, resulting in an increase in organ failure. Legrand et al. reported higher CVP in septic patients under mechanical ventilation, leading to a higher frequency of AKI [9]. A recent meta-analysis confirmed this association [1].

The portal vein (PV) pulsatility index (PI) has been developed and described as a noninvasive alternative to measuring CVP to monitor venous congestion [10]. The PI is defined as the relative difference between the maximal and minimal portal blood flow velocity. A significant amount of data has evidenced the association between a high PI and venous congestion. More recently, studies of postoperative care have reported that PI values > 50% are associated with adverse outcomes, and more specifically, AKI [11,12,13]. Spiegel and al. reported that a PI > 30% was associated with renal injury when assessed in a nonspecific population of critically ill patients [14].

There are currently no data on variations in the PI during mechanical ventilation. Clinical data confirmed that mechanical ventilation is a risk factor for AKI in a recent meta-analysis [15]. As high ventilator pressure may increase CVP, and CVP seems correlated to PI, the PI could be a useful tool to monitor venous congestion at the bedside during mechanical ventilation [16,17,18,19].

We aimed to assess how an incremental PEEP could influence PI supposing a backward transmission of PEEP in the venous system. We assume that a high PEEP leads to an increase in the pulsatility index. The higher the PEEP the higher the PI.

## 2. Materials and Methods

### 2.1. Ethical Approval

The study protocol was approved by the local independent ethics committee (Comité de Protection des Personnes Nord-Ouest II, Amiens, France; reference TB/LR/2016-19) on 24 March 2016. The present report was drafted in accordance with the STROBE statement for cohort studies [20]. The study complied with the Declaration of Helsinki on ethical principles for medical research involving human subjects. All patients received written information on the study and gave their verbal consent to participate.

### 2.2. Study Participants

We conducted a prospective monocentric study at Amiens University Hospital in the cardiothoracic unit. The main inclusion criteria were patients under mechanical ventilation and under sedation following cardiac surgery. The exclusion criteria were an age < 18 years, atrial fibrillation, the presence of a pacemaker, acute circulatory failure requiring a vasoactive drug, poor echogenicity, acute respiratory distress syndrome, a medical history of cirrhosis or chronic hepatic disease, and right heart dysfunction.

### 2.3. Portal Flow Pulsatility Fraction Measurement

Ultrasound assessments were performed using transthoracic echography (TTE) by physicians with training in critical-care ultrasound. Portal blood assessment was performed by the same physician to prevent inter-observer variations and the investigators performing the ultrasound measurements were not involved in the clinical care of the patients. TTE was performed at the bedside during the post-operative period and for each stepwise, we waited for 5 min after PEEP increment. The PI was averaged from five measurements (regardless of the respiratory cycle).

The assessment consisted of the pulsed-wave Doppler evaluation of the PV in the liver hilum. Doppler evaluation of the PV with TTE was performed as described by Denault et al. [21]. The maximum velocity (VMAX) and minimum velocity (VMIN) of the PV was measured in pulsed Doppler mode. The portal flow PI was calculated using the following formula PI = (VMAX − VMIN)/(VMax). PV hypertension can be detected with a PI ≥ 30% [22].

### 2.4. Hemodynamic Parameters

Several other echocardiographic and hemodynamic parameters were recorded. Stroke volume (SV) was measured by TTE (CX50 Ultrasound System and an S5-1 Sector Array Transducer, Philips Medical System, Suresnes, France), which was performed by a single experienced physician. The left ventricular ejection fraction (LVEF), end-systolic volume (ESV), and end-diastolic volume (EDV) were measured using Simpson’s biplane method with a four-chamber view. The diameter of the left ventricular outflow tract (LVOT) was measured in a long-axis parasternal view upon patient inclusion. The aortic surface area (SAo, in cm²) was calculated as π × LVOT2/4. The aortic velocity-time integral (VTIAo) was measured by pulsed Doppler and a five-chamber apical view. SV (mL) was calculated as VTIAo × Sao and CO (in L·min^−1^) as an SV × heart rate (HR). The right ventricular ejection fraction (RVEF) was measured using Simpson’s biplane method on a four-chamber view. The tricuspid annular systolic velocity at the lateral wall (Sr(t)) and M-mode annular systolic excursion plane (tricuspid annular plane systolic excursion (TAPSE)) were measured by placing the tissue-Doppler pulse wave and M-mode sample volume at the level of the basal tight ventricular free wall. The systolic–diastolic (S/D) ratio was calculated as S/D = maximum systolic velocity [cm/sec]/maximum diastolic velocity (cm/s). In normal subjects, the S wave is wider than the D wave, with an S/D ratio > 1. To assess the S/D ratio, we performed a hepatic-vein Doppler. The S wave is a result of ventricular systolic flow and shows a negative systolic wave. The D wave is a result of passive filling of right ventricle. Right ventricular dysfunction was defined as an alteration of systolic function parameters with one of the following: S wave < 10 cm/sec, TAPSE < 16 mm cm, FeVD < 45%, RV fractional area change <35%, or presence of right ventricular dilatation [5].

The mean echocardiographic parameters were calculated from five measurements (regardless of the respiratory cycle). Data were acquired and off-line images were reviewed by the operator blind to the PV measurements.

### 2.5. Intervention

Operative and post-operative interventions were standardized for all patients [23]. Sedation during the intervention was maintained by continuous infusion of propofol. All patients underwent mechanical ventilation in volume-controlled mode, with the tidal volume set to 7–8 mL kg^−1^ ideal body weight [24]. Ventilator settings (oxygen-inspired fraction, tidal volume, respiratory rate) were not modified during the study period. We then performed a PEEP increment trial from 0 to 15 cmH_2_O by increments of 5 cmH_2_O. Echocardiography, PV flow (mL min^−1^), portal sectional area (m^2^), PI (%), and hepatic venous blood velocity were assessed at baseline and at PEEP 5, 10, and 15. Baseline and post-PEEP increment data were collected, along with the following experimental data: systolic arterial pressure, diastolic arterial pressure and mean arterial pressure, and heart rate. We also collected the following demographic data for all patients: weight (kg), height (m), and body mass index (BMI), expressed as the ratio of the weight to height^2^ (kg m^−2^).

### 2.6. Statistical Analysis

Data are expressed as means ± standard deviations (SDs), medians (interquartile ranges), or numbers (percentages), as appropriate. Variables were compared using Wilcoxon–Mann–Whitney, chi-2, or Wilcoxon rank sum tests, as appropriate. Correlations between SV, PI, and portal blood flow were assessed using the non-parametric Spearman correlation test. A receiver operating curve was generated to assess the predictive value of the portal flow and the PI to decrease SV following PEEP incrementation. All statistical analyses were performed using IBM SPSS software (SPSS, version 24, IBM, New York, NY, USA). The threshold for statistical significance was set to *p* < 0.05.

## 3. Results

**Figure 1:**
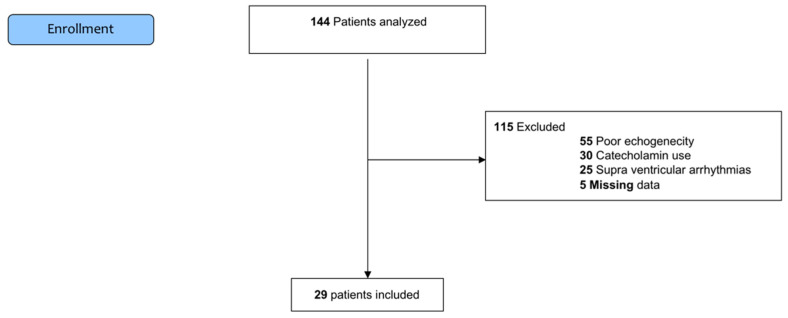
Flowchart.

Demographic Data (Table 1 and Figure 1).

In total, 144 patients were screened from February 2018 to March 2019 and 29 were included, as presented in the flow chart (Figure 1). Overall, 115 patients were excluded. Among these, 55 patients were excluded for incomplete echocardiographic data in relation to poor echogenicity, 30 patients were excluded for catecholamine use, 25 patients were excluded for supraventricular arrhythmia, and 5 patients were excluded for missing data. The mean age was 69 (61–77) years, with a BMI of 27.5 (24.4–30.1) kg m^−2^. At inclusion, no patients showed acute circulatory failure or criteria of acute respiratory syndrome. Baseline data are presented in Table 1.

**Figure 2:**
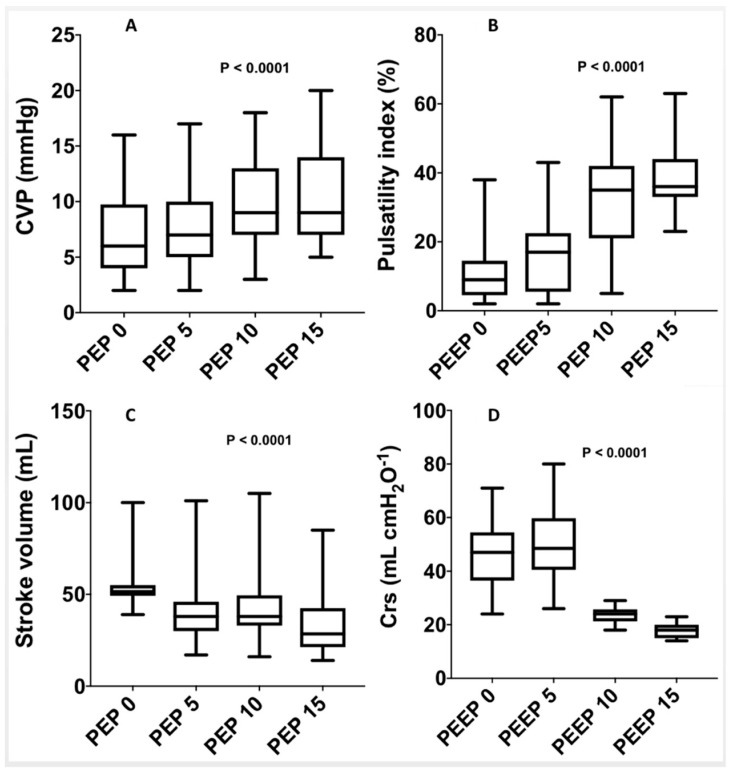
Systemic and hepatic hemodynamic changes after PEEP incrementation: (**A**) changes in CVP with changes in PEEP; (**B**) changes in the pulsatility index (%) with changes in PEEP; (**C**) changes in stroke volume with changes in PEEP; (**D**) changes in Crs with changes in PEEP. Crs: compliance of the respiratory system; CVP: central venous pressure; PEEP: positive end-expiratory pressure.

Hemodynamics and Echocardiographic Changes after PEEP Incrementation (Table 2 and Figure 2).

Systolic, diastolic, and mean arterial pressure were significantly lower after PEEP incrementation. SVs were also significantly lower after PEEP incrementation, with 52 mL (50–55) at PEEP 0 cmH_2_O and 30 mL (25–45) at PEEP 15 cmH_2_O, (*p* < 0.0001). In addition, the cardiac index decreased from 2.2 L·min^−1^ (2.07–2.42) to 1.2 L·min^−1^ from PEEP 0 to PEEP 15 (*p* < 0.0001).

At baseline, the CVP was 6 (4–10) cmH_2_O and significantly increased for each PEEP increment, with CVPs at PEEP 5, 10, and 15 cmH_2_O of 7 (5–10), 11 (8–13), and 12 (9–15) cmH_2_O, respectively, *p* < 0.001. The S/D ratio decreased, from an S/D ratio of 2.0 (1.30–2.99) at PEEP 0 cmH_2_O to 1.08 (0.92–1.34) at PEEP 15 cmH_2_O (*p* < 0.0001). We observed a nonsignificant decrease in the right ventricular systolic parameters with the TAPSE and S wave.

Portal Hemodynamic Changes after PEEP Incrementation (Table 2 and Figure 2).

PI significantly increased with PEEP incrementation, from 9% (5–15) at PEEP 0 cmH_2_O to 15% (5–22) at PEEP 5 cmH_2_O, 34% (23–44) at PEEP 10 cmH_2_O, and 45% (25–49) at PEEP 15 cmH_2_O (*p* < 0.001).

Relationship between Portal Flow Pulsatility Fraction (PI) and Variations in Stroke Volume (SV) (Table 3).

SV was negatively correlated with the PI (Rho = −0.360, *p* = 0.084). There was no correlation between the SV and mean arterial pressure, regardless of the PEEP level.

## 4. Discussion

Our findings suggest that, during mechanical ventilation, an increase in PEEP is associated with increases in CVP and the PI: a higher PEEP indicates a higher PI. We observed the dynamic variation of the PI with the increase in PEEP. The PI started from a very low variation, to values over 50% when the PEEP was at 15 cmH_2_O.

The normal waveform of the portal vein is a continuous monophasic flow above baseline with minor variations. As venous congestion worsens, the portal flow becomes pulsatile. In the case of the transmission of thoracic pressures to the right atrium, we observed an increase in the right atrial pressure and therefore a decrease in the venous return gradient leading to congestion (increase in IVC diameter as well as a decrease in the collapsibility index and a decrease in the S/D ratio). The pressure variations in the right atrium during the cardiac cycle were transmitted through the noncompliant venous system and then the PI increased. We observed a decrease in cardiac output (associated with the increase in PEEP), which does not explicate the increase in PI [5].

A previous study performed a single assessment of the PI during an ICU stay and concluded that a single measurement could predict adverse outcomes [22]. We performed several measurements at different time points. PEEP incrementation was chosen as an interventional maneuver to change the right heart load conditions. We hypothesized that the PI is a dynamic marker, allowing the monitoring of the effect of mechanical ventilation on venous return and thus congestion. Mechanical ventilation is a risk factor of AKI in critically ill patients [15]. Among possible explanations, venous congestion is highly probably. Increased CVP results in a decrease in the drive pressure between the systemic vein compartment and the right atrium. This may compromise the perfusion of organs, such as the kidneys. This hypothesis is supported by studies in which the CVP was higher in patients with mechanical ventilation and AKI [10]. Furthermore, a meta-analysis confirmed that CVP is an independent factor of AKI [1].

Right-side pressure and CVP have been shown to be associated with AKI and worse outcomes in the ICU [25]. In the present study, PI correlated with CVP. CVP is regularly used in the ICU but is subject to numerous technical limitations and interobserver variability [26]. Furthermore, no CVP cut-off has been shown to predict the complications related to venous congestion. However, PI could represent a dynamic point-of-care ultrasound marker for the assessment of elevated venous pressure and significant organ congestion [11]. Point-of-care ultrasound (POCUS) allows physicians to assess venous congestion using venous waveforms of large veins, including the PV [27]. According to the vascular system complaint, the increase in volume, associated with congestion, leads to an increase in pressure and therefore in curves. A recent study proposed a venous excess ultrasound grading system using a combination of multiple POCUS markers to identify significant venous congestion [27]. There is no consensus on what constitutes a high PI value, although most studies have considered a PI value of >50% to be high. PI has been shown to be correlated with right atrial pressure [10]. Following cardiac surgery, there is a significant association between PI and RV dysfunction [12,21].

Our study had several limitations. We did not assess AKI. Thus, we could not show a relationship between PI and AKI, but several publications have reported an association between AKI and PI through venous congestion [11,13]. Patients with high PV pulsatility have been shown to be more likely to develop AKI following cardiac surgery [12]. Ours was a proof-of-concept study to confirm the interaction between mechanical ventilation and the kidney through the venous compartment. In addition, our study offers preliminary data to conduct a larger intervention study and monitor the PI in mechanically ventilated patients—particularly in ARDS, in which high PEEP is applied. Another limit was the small sample size and the absence of data on the fluid balance. We thus voluntarily chose one specific postoperative population to have the most homogenous baseline data possible before inclusion. A high level of PEEP is not recommended in all patients; here, we used it during postoperative recruitment maneuvers in order to increase right atrial pressure and thus determine the variation of PI [28]. French recommendations on enhanced recovery after cardiac surgery recommend systematic recruitment maneuvers postoperatively [29]. During mechanical ventilation, monitoring PI could be an interesting approach.

## 5. Conclusions

PI appears to be a dynamic marker of the interaction between mechanical ventilation and right heart pressure after cardiac surgery. Future studies are needed to clarify the benefit of routine monitoring of the PI during mechanical ventilation to prevent organ failure.

## Figures and Tables

**Table 1:** Baseline demographic data.

Variables	Overall Population (*n* = 29)
Age—years	69 (61–77)
Male sex—n (%)	16 (55)
BMI—kg m^−2^	27.5 (24.4–30.1)
Surgery type—n (%)	
Valve replacement	15 (52)
Isolated CABG	5 (17)
Mitral valve surgery	7 (24)
Combined surgery	1 (3)
Other	1 (3)
Medical history n (%)	
Hypertension	22 (76)
Diabetes	8 (21)
Dyslipidemia	13 (45)
Smokers	8 (28)
Baseline LVEF (%)	63 (55–66)
Baseline TAPSE (mm)	21 (16–26)
Respiratory characteristics at baseline	
Lung compliance, cmH_2_O^−1^	49 (41–60)
Tidal volume, mL k^−1^	6.4 (5.5–6.8)
PaO_2_/FiO_2_	440 (360–510)
Respiratory rate (/min)	15 (14–16)
Driving pressure; cmH_2_O	10 (7–11)
ASA score (II/III) (n)	3/26
Logistic Euroscore—%	6 (4–10)
SAPS II	28 (24–40)
Biologic data at baseline	
Temperature—°C	36.3 (36.2–36.6)
pH	7.38 (7.34–7.40)
PaCO_2_—mmHg	40 (36–44)
Lactate—mmol·L^−1^	1.3 (1.1–1.5)

Values are presented as mean ± SD or numbers (%). ASA: American Society of Anesthesiologists; BMI: body mass index expressed as the ratio of weight to height; CABG: coronary artery bypass graft; LVEF: Left ventricular ejection fraction; SAPS: simplified acute physiology score. TAPSE: Tricuspid annular plane systolic excursion.

**Table 2:** Evolution of the clinical, echocardiographic, and hepatic parameters after PEEP incrementation.

Variables	PEEP 0	PEEP 5	PEEP 10	PEEP 15	*p* Value
Clinical parameters					
Lung compliance (mL/cmH_2_O)	44 (36–53)	48 (40–59)	24 (15–20)	18 (15–20)	<0.0001
HR (bpm)	80 (65–93)	76 (63–126)	82 (61–93)	82 (90)	0.089
SAP (mmHg)	115 (109–130)	119 (101–126)	112 (92–133)	97 (75–117)	<0.0001
DAP (mmHg)	62 (54–71)	55 (49–67)	57 (49–65)	51 (45–65)	<0.0001
MAP (mmHg)	79 (72–90)	79 (66–87)	77 (60–86)	64 (56–81)	0.008
PPV (%)	10 (8–13)	12 (8–15)	15 (8–20)	16 (11–22)	<0.0001
CVP (mmHg)	6 (4–10)	7 (5–10)	11 (8–13)	12 (9–15)	0.002
Echocardiographic parameters					
SV (mL)	52 (50–55)	39 (31–46)	35 (39–36)	30 (25–45)	<0.0001
CI (L min^−1^ m^−2^)	2.2 (2.07–2.42)	1.7 (2.12)	1.6 (1.4–2.2)	1.2 (1.0–1.8)	<0.0001
TAPSE (mm)	10 (8–12)	7 (5–12)	8 (7–10)	7 (4–8)	0.125
S wave (m/s)	8.7 (11.6–12.6)	7.3 (6.4–7.8)	7.3 (6.4–7.7)	5.6 (4.3–6.7)	0.194
IVC collapsibility (%)	22 (10–32)	30 (24–38)	13 (6–18)	9 (4–19)	<0.001
IVC max (mm)	15 (14–19)	20 (14–22)	19 (17–21)	19 (17–21)	<0.001
IVC min (mm)	12 (11–14)	17 (13–20)	17 (14–19)	18 (15–20)	<0.001
Hepatic hemodynamics					
S/D ratio	2.0 (1.30–2.99)	1.74 (1.22–1.88)	1.43 (1.19–1.56)	1.08 (0.92–1.34)	<0.0001
Portal vein diameter (cm)	0.49 (0.44–0.58)	0.55 (0.51–0.61)	0.48 (0.41–0.57)	0.55 (0.47–0.64)	0.081
The portal vein pulsatility index (%)	9 (5–15)	15 (5–22)	34 (23–44)	45 (25–49)	<0.0001

CI: cardiac index; CVP: central venous pressure; DAP: diastolic arterial pressure; HR: heart rate expressed in beats per minutes (bpm); MAP: mean arterial pressure; PEEP: positive end-expiratory pressure; PPV: pulse pressure variation; SAP: systolic arterial pressure; SV: stroke volume; TAPSE: tricuspid annular plane systolic excursion. Pulsatility index (%) was expressed as follows: 100 × (maximal velocity − minimal velocity)/maximal velocity. Comparisons were performed using Wilcoxon rank sum test.

**Table 3:** Correlations between variations in the portal flow pulsatility fraction and stroke volume (SV) following PEEP incrementation.

Variables	SVPEEP 0	SVPEEP 5	SVPEEP 10	SVPEEP 15
PI	−0.334 *	−0.350 *	−0.123 *	−0.318 *
MAP	0.126	0.178	0.237	0.378
S wave	0.440 *	0.418 *	0.479	0.425 *
TAPSE	0.185	0.654 *	0.382 *	0.393 *

MAP: mean arterial pressure; PEEP: positive end-expiratory pressure; PF: portal flow pulsatility fraction; SV: stroke volume; TAPSE: tricuspid annular plane systolic excursion. Portal vein pulsatility index (PI) (%) was expressed as follows: 100 × (maximal velocity − minimal velocity)/maximal velocity. Spearman’s correlation analysis was used to identify the correlations. *: *p* value < 0.05.

## Data Availability

Data are available on request according to restrictions (e.g., privacy or ethical).
